# Editorial: Endocrinology, lipids, and disease: unraveling the links

**DOI:** 10.3389/fendo.2025.1687649

**Published:** 2025-09-04

**Authors:** Ashutosh Prince, Prem Prakash Kushwaha, Mathias Blüher, Dirk Müller-Wieland, Robert Kiss

**Affiliations:** ^1^ Department of Biological, Geological and Environmental Science, Cleveland State University, Cleveland, OH, United States; ^2^ Centre of Gene Regulation in Health and Disease, Cleveland State University, Cleveland, OH, United States; ^3^ Helmholtz Institute for Metabolic, Obesity and Vascular Research (HI-MAG) of the Helmholtz Zentrum München at the University of Leipzig and University Hospital Leipzig, Leipzig, Germany; ^4^ Klinik für Kardiologie, Angiologie und Internistische Intensivmedizin, Universitätsklinikum RWTH Aachen, Aachen, Germany; ^5^ Research Institute of the McGill University Health Centre, McGill University, Montreal, QC, Canada

**Keywords:** lipocrinology, endocrine regulation, lipid-based biomarkers, metabolic syndrome, extracellular vesicles, triglyceride-glucose (TyG) index

## Introduction

The conventional view of lipids as a structural components or energy reservoirs have recently gained a more dynamic paradigm. Lipids are now a central molecular regulator in endocrine biology—act as active signaling molecules that profoundly shape hormone synthesis, secretion, and systemic action. This conceptual shift is at the heart of lipocrinology: the integration of lipid metabolism with endocrine function. The eighteen contributions assembled in this Research Topic, *Endocrinology, Lipids, and Disease: Unraveling the Links*, collectively advance this perspective, interweaving together key findings from molecular biology, systems-level pathophysiology, clinical diagnostics, and therapeutic innovation. This Research Topic moved beyond isolated observations to construct a cohesive, multi-scale narrative of how the intricate crosstalk between lipids and hormones governs metabolic health and disease.

## Foundational mechanisms: from cellular machinery to systemic networks

At the most fundamental level, endocrine function is dependent on lipid biology. The mini-review by Aderhold and Alexaki provides a coherent overview of this within the adrenal gland, illustrating how specific lipids—including cholesterol, diacylglycerol, and phosphoinositides—are not just substrates but critical regulators of both steroidogenesis in the cortex and catecholamine exocytosis in the medulla. This work establishes the lipid-mediated orchestration of hormonal relay as a core cellular process.

Expanding from the cell to the system, the perspective by Łukowski et al. described a transformative model for type 1 diabetes, a classically defined autoimmune endocrinopathy. They proposed hypothesis at the Endocannabinoidome-Microbiota (ECBoM) axis as a systemic disorder arising from a dysfunctional interplay between gut dysbiosis, lipid-derived endocannabinoid signaling, and immune dysregulation. This perspective, which emphasizes a network-based approach, redirects the cause of the condition from the pancreatic islet to a more comprehensive disruption in metabolic and immune balance. It implies that the autoimmune disorder is a secondary effect resulting from an imbalance among lipids, microbes, and the immune system on a systemic level.

## Advancing the diagnostic frontier with integrated biomarkers

A prominent theme emerging from this Research Topic is the validation of biomarkers by apprehending the functional states of metabolic dysregulation. The triglyceride-glucose (TyG) index, a substitute for insulin resistance, is a key example. Zeng et al. demonstrate its potent predictive value in a cohort of patients with acute myocardial infarction, showing that a high TyG index independently predicts the formation of left ventricular aneurysms and cardiac death. Complementing this, Yan et al. employed machine learning algorithms on a large NHANES dataset, identifying the TyG index and its derivatives as the most powerful predictors of all-cause and cardiovascular mortality among eight lipid-related indicators in individuals with diabetes or prediabetes.

This principle of integrated risk assessment extends to other markers. Zhou et al. reveal that impaired sensitivity to thyroid hormones, even in euthyroid individuals, is strongly associated with the severity of metabolic syndrome, highlighting that tissue-level hormone resistance is a metabolic disruptor. Markers of visceral adiposity also show significant prognostic power. Pan et al. report a robust, L-shaped inverse correlation between the Lipid Accumulation Product (LAP) and osteoporosis in American adults, uncovering a critical lipid-bone axis. Similarly, work by Ding et al. associates perirenal fat thickness—a specific visceral fat depot—with hypertension and an elevated 10-year cardiovascular disease risk, emphasizing the endocrine influence of ectopic fat on vascular tone. Further illuminating the complexity of multi-organ damage, Jin et al. (Xue et al.) demonstrate how Advanced Glycation End Products (AGEs) interact with metrics like the TyG-BMI index to predict the risk of diabetic nephropathy, showcasing the synergy between glycation and lipotoxicity in driving renal disease.

## Lipids and hormones in concert: diverse organ impacts of lipocrinology

The research presented illustrates that disruptions in the lipid-endocrine axis are not confined to classic metabolic organs but have far-reaching systemic consequences. In the liver, Meyer et al. uncover a mechanistic basis for sex differences in metabolic-associated steatotic liver disease (MASLD). Their preclinical model showed that female mice are protected from severe liver damage due to preferential lipid partitioning into adipose tissue, a process modulated by the differential expression of estrogen receptors that underscores the powerful influence of the hormonal milieu on organ-specific disease susceptibility. In reproductive health, Tu and Fang utilize integrative bioinformatics to link endometriosis directly to fatty acid metabolism. They identify six hub genes, including *PTGS2* and *ACSL4*, that resides at the nexus of lipid metabolism and inflammation, providing a molecular framework for understanding endometriosis as a metabolic-inflammatory disease and suggesting novel therapeutic targets. The systemic reach extends to the neuro-endocrine axis, where Liu et al. identify shared genetic variants and biological pathways between obesity and depression, particularly those related to inflammation and metabolic regulation. This finding points to a common genetic architecture underlying these frequently co-occurring conditions. Furthering this connection, Su et al. reported from a large prospective cohort that sufficient serum 25-hydroxyvitamin D levels are associated with a nearly 50% lower risk of sleep disorders in individuals with prediabetes or diabetes, highlighting the role of this fat-soluble hormone in regulating central processes beyond calcium homeostasis.

## Therapeutic horizons: modulating lipid-endocrine pathways

Moreover, a deeper mechanistic understanding must translate into improved therapies. Several articles in this Research Topic explore interventions that target the lipid-endocrine network. A study by a separate Leng et al. cohort demonstrates that Ebenatide, a GLP-1 analogue, not only improves glycemic indices but also significantly reduces the TyG index and fat mass in patients with type 2 diabetes, showing the efficacy of hormonal agents in modifying both glucose and lipid pathways.

The therapeutic potential of natural compounds is also highlighted. Zong et al. review the evidence for lactoferrin, a natural protein, as a pleiotropic agent that can alleviate insulin resistance and inflammation via multiple signaling pathways, including PI3K/Akt. In a preclinical model of non-alcoholic steatohepatitis (NASH), Liu et al. showed that active vitamin D3 mitigates liver damage by modulating fatty acid metabolism, oxidative stress, and inflammation, positioning it as a potent metabolic regulator. Beyond pharmacology and nutraceuticals, *Zhang* et al. present a meta-analysis indicating that acupuncture can significantly improve glycemic and triglyceride profiles in patients with T2DM, suggesting that non-drug modalities can effectively restore lipid-hormonal balance.

## Synthesis: embracing complexity and future directions

The ultimate understanding offered by this Research Topic highlights the intricate and non-linear characteristics of these biological systems. The work by Yan et al., for instance, describes a U-shaped association between serum uric acid and metabolic risk, challenging simplistic linear assumptions and revealing that both low and high levels of a metabolite can be associated with pathology. This reflects the importance of maintaining metabolic homeostasis within a specific physiological range. The identification of shared genetic loci for obesity and depression by Tian et al. further strengthens that these clinical phenotypes are the emergent properties of embedded, interconnected biological networks.

Taken together, these eighteen articles chart the course of a field moving decisively toward an integrated, systems-level perspective. They firmly establish that lipids are not passive molecular integrity but active endocrine modulators that integrate cellular machinery, hormonal feedback loops, and systemic networks. The consistent outperformance of composite biomarkers like the TyG index and LAP signals a necessary evolution in clinical diagnostics, while therapeutic successes with agents like GLP-1 analogues and even non-traditional interventions like acupuncture highlight the promise of targeting the lipid-endocrine axis directly. This Research Topic solidifies *lipocrinology* as an essential conceptual framework, driving the field toward a more holistic and summarized approach to understanding the endocrine and metabolic diseases.

